# Application of e-PTFE Frontalis Suspension in the Treatment of Congenital Ptosis in Children

**DOI:** 10.3389/fsurg.2022.904307

**Published:** 2022-05-16

**Authors:** Ling Ma, Lei Zhang, Zhen Liu, Dandan Wang, Yibao Li, Chengyue Zhang

**Affiliations:** ^1^ Department of Ophthalmology, Pediatric Hospital Affiliated to Fudan University, Anhui Hospital, Anhui Children’s Hospital, Hefei, China; ^2^ Department of Ophthalmology, Fuyang People’s Hospital, Fuyang, China; ^3^ Department of Ophthalmology, Beijing Children’s Hospital, Beijing, China

**Keywords:** congenital ptosis, e-PTFE, frontalis suspension, frontalis flap suspension, children

## Abstract

**Purpose:**

Analysis of the value of expanded polytetrafluoroethylene (e-PTFE) frontalis suspension applied to children with congenital ptosis.

**Methods:**

Eighty clinical cases of children with congenital ptosis from October 2019 to October 2021 were randomly selected from our hospital. All children were divided into the observation group (*n* = 44) treated with e-PTFE frontalis suspension and the control group (*n* = 36) treated with frontalis flap suspension according to the treatment procedure. Comparison of eyelid condition [palpebral fissure height, margin reflex distance (MRD), eyelid closure time], ocular surface status [corneal fluorescein staining (CFS) score, tear film breakup time (TBUT), surgical eye lacrimal river height (LRH), sehirmer test I (STI)], frontal muscle strength of affected side, cosmetic results and complications in both groups at 1, 6 and 12 months postoperative follow-up.

**Results:**

At 1, 6 and 12 months after surgery, there was no significant difference in terms of palpebral fissure height and MRD between both groups (*p* > 0.05); After surgery, the eyelid closure time was shorter in the observation group than in the control group (*p* < 0.05). At 1, 6 and 12 months after surgery, the CFS scores were lower in the observation group than in the control group (*p* < 0.05); At 6 and 12 months after surgery, the TBUT was longer and the surgical eye LRH was higher in the observation group than in the control group (*p *< 0.05); At 1, 6, and 12 months after surgery, there was no significant difference in STI between both groups (*p* < 0.05). At 1, 6 and 12 months after surgery, the frontal muscle strength of affected side was higher in the observation group than in the control group (*p* < 0.05). At 1, 6, and 12 months after surgery, there was no significant difference in cosmetic results between both groups (*p* > 0.05). The overall complication rate in the observation group (6.82%) was lower than that in the control group (25.00%) (*p* < 0.05).

**Conclusion:**

The surgical and cosmetic results of e-PTFE frontalis suspension and frontalis flap suspension applied to congenital ptosis are comparable, but the former has the advantage of faster postoperative recovery, better ocular surface status, less frontali muscle strength damage and fewer complications.

## Introduction

With the exclusion of the effects of the frontalis muscle, ptosis can be diagnosed when the upper eyelid margin covers the upper edge of the cornea by more than 2 mm when the eyes are open and level-viewing, which is a common eyelid deformity caused by weak or defective upper eyelid muscle development or oculomotor and cervical sympathetic insufficiency (1, 2). The child has partial or complete loss of levator muscle function, and partial or complete ptosis of the upper eyelid obscuring the pupil. In mild cases, it affects the appearance and has a negative impact on psychological and personality development during the growth of the affected child; in severe cases, the pupil is obscured, affecting visual development and leading to form deprivation amblyopia (prevalence up to 20%–30%) with or without refractive error, which can also lead to abnormal development of the neck muscles and cervical spine (3, 4). Especially in patients with monocular disease, the degree of amblyopia is deeper and the correction is more difficult, and active surgical treatment, refractive correction, and amblyopia treatment are required during the critical period of visual development (3–6 years old) of children (5).

Among the current domestic and international corrective procedures, frontalis suspension is the generally accepted type of surgery. It moves the pedicled frontalis tissue flap directly down the eyelid to fix and lift the upper lid. Compared with the clinically common levator muscle shortening, the frontalis muscle flap has better elasticity and greater muscle strength, which is conducive to the adjustment of the tarsal radian in clinical operations, and the postoperative aesthetic effect of patients is more durable, but the application effect of different materials during the operation is still controversial (6). Expanded polytetrafluoroethylene (e-PTFE) is a new type of medical polymer material with good biocompatibility, convenient material extraction, good flexibility, high strength, good operating feel and minimally invasive effect (7, 8). Its unique microporous structure allows human tissue cells and blood vessels to grow in and form tissue connections without causing tissue reactions. It is now widely used in pediatric surgery to adapt to the growth of children’s tissues (9). This study compares the near and long-term efficacy and safety of e-PTFE frontalis suspension with frontalis flap suspension in children with congenital ptosis, with the aim of providing a relevant reference for the use of e-PTFE frontalis suspension in children with congenital ptosis.

## Materials and Methods

### Research Object

Eighty clinical cases of children with congenital ptosis from October 2019 to October 2021 were randomly selected from our hospital. All children were divided into the observation group (*n* = 44) treated with e-PTFE frontalis suspension and the control group (*n* = 36) treated with frontalis flap suspension according to the treatment procedure. Inclusion criteria: age 1–6 years old; Meet the 2017 “Expert Consensus on Diagnosis and Treatment of Ptosis” (10) for the diagnosis and classification criteria of moderate and severe ptosis: When children open their eyes and look straight ahead, those with ptosis covering more than 4–6 mm above the cornea and the amount of ptosis >2–≤4 mm are considered moderate; those with ptosis that cover the upper part of the cornea by >6 mm and the amount of ptosis >4 mm are considered severe; congenital ptosis; unilateral disease; those who had completed routine ophthalmic examination before surgery; preoperative Bell’s sign positive; those who presented with symptoms such as visual impairment and pupil narrowing; those with poor upper eyelid levator function or whose upper eyelid levator structure had been damaged; the family members of the children all signed and agreed to obey the treatment. Exclusion criteria: bilateral disease; ptosis caused by acquired factors, such as surgery, trauma, tumor invasion, levator aponeurosis hole or rupture, oculomotor nerve or oculomotor nerve branch paralysis, myasthenia gravis, myotonia syndrome, progressive muscular dystrophy; persons with other ophthalmic diseases such as corneal disease, glaucoma, ocular trauma, extensive extraocular muscle fibrosis syndrome, etc.; persons with systemic diseases; persons with abnormal intellectual development, mental disorders, etc. that prevented them from cooperating with the study. Comparing the general conditions of age, gender, enrolled affected eyes, and degree of ptosis in both groups, *p* > 0.05, which available for comparative study, as shown in **Table 1**.

**Table 1:** Comparison of general conditions of two groups.

Itmes	Observation group (*n* = 44)	Control group (*n* = 36)	*t/χ*^2^ value	*p* value
Age ($\bar{x}\pm s$, years old)	3.64 ± 1.01	3.61 ± 0.96	0.135	0.893
Gender (*n*, %)			0.013	0.910
Male	25 (56.82)	20 (55.56)		
Female	19 (43.18)	16 (44.44)		
Enrolled affected eyes (*n*, %)			0.037	0.848
Left eye	35 (79.55)	28 (77.78)		
Right eye	9 (20.45)	8 (22.22)		
Degree of ptosis (*n*, %)			0.080	0.777
Moderate	28 (63.64)	24 (66.67)		
Severe	16 (36.36)	12 (33.33)		

### Surgery Methods

Both groups of children underwent routine ophthalmological examination after hospitalization. Including fundus, anterior segment and refractive status, frontalis muscle strength, eyelid closure, levator muscle strength, margin reflex distance (MRD), Bell’s sign, etc. on the basis of it.

In the observation group, e-PTFE frontalis suspension was applied to treat: The child was placed supine, and after successful general anesthesia with sevoflurane inhalation, a double eyelid incision was made at 2–3 mm of skin from the upper eyelid margin, and a 0.5 cm skin incision was made on the inner and outer 1/3 of the brow arch and 2 cm above the brow arch. The subcutaneous tissue and the orbicularis oculi muscle were separated from the incision to expose the tarsal plate. The e-PTFE material was secured to the tarsus 3 mm from the upper lid margin using 6-0 polypropylene synthetic sutures. A 0-gauge curved needle was used to tract the e-PTFE material through a subcutaneous tunnel to reach the deeper tissue under the arch of the eyebrow. The height of the eyelid was adjusted, the suspension material was ligated and buried under the frontalis muscle, and Frost protective suture was made on the lower eyelid margin.

In the control group, the frontalis flap suspension was applied to treat: The child was placed supine, and after successful general anesthesia with sevoflurane inhalation, a double eyelid line was drawn at a distance of 2–3 mm from the eyelid margin. The skin at the double eyelid line was incised, separated from the front and upward of the orbital septum under the orbicularis oculi muscle, the frontalis muscle flap was pulled down, and 3-needle mattress sutures were placed with tarsus by 4-0 mousse thread. The height of the eyelid margin was adjusted and the suture was ligated, and the Frost suture was placed on the lower eyelid.

In both groups, pressure bandages were applied to the operated eye and forehead to prevent bleeding and hematoma, and antibiotic eye ointment was applied to the conjunctiva. The corneal and postoperative swelling were observed after 24 h, and any problems discovered were treated promptly and the pressure bandage was removed after 48 h. Eye drops were used continuously during the day, and chlortetracycline eye ointment was applied at night. The sutures were removed 7 d after surgery, and the frontalis muscle exercise was performed.

### Observation Index

(1) Comparison of eyelid status, including palpebral fissure height, MRD, and eyelid closure time, between the two groups of children at 1, 6, and 12 months postoperative follow-up.
(2) Comparison of the ocular surface status of children in the two groups at 1, 6 and 12 months postoperative follow-up. Including corneal fluorescein staining (CFS) score (the cornea was divided into 4 quadrants, each quadrant was assigned a score of 0–3 according to the no, light, moderate and severe degree of staining, with a total score of 0–12), tear film breakup time (TBUT) (after fluorescein staining of the tear film, the subject looked forward flatly and kept the eyes open after blinking several times, under the cobalt blue light of the slit lamp microscope, the time interval for the appearance of the first dry spot on the tear film surface was observed with a wide slit lamp strip, that is, the tear film breakup time, the normal value is 10–45 s), surgical eye lacrimal river height (LRH) (after fluorescein staining, the tear level at the junction of the light band projected on the surface of the cornea and conjunctiva and the light band of the lower eyelid margin under the slit lamp microscope, the normal tear river section is convex, with a height of 0.3–0.5 mm), sehirmer test I (STI) (5 min wet length and the time to 10 mm wet length were recorded using Whatmann No. 41 filter paper provided by BauschLomb Inc., USA).
(3) Comparison of the frontal muscle strength of the affected side in the two groups of children at 1, 6 and 12 months postoperative follow-up. Assessment by manual muscle testing (MMT), verbal instructions and actions, such as frowning, were demonstrated to the child, the completion of the expression muscle was observed and compared with the normal side. The MMT muscle strength grading criteria are shown in **Table 2**.
(4) Comparison of the cosmetic results of the two groups of children at 1, 6 and 12 months postoperative follow-up. Including three test items: double eyelid incision, eyelid shape, and double eyelid height symmetry, the specific grading standards and scores of each item are shown in **Table 3**.
(5) Comparison of postoperative complications in the two groups of children, including exclusion reactions (nodules, infections, granulomas), upper eyelid inversion trichiasis, exposure keratitis, and incomplete eyelid closure.

### Statistical Methods

SPSS 22.0 software was applied, and the measurement data were expressed as mean ± standard deviation $\left(\bar{x}\pm s\right)$ and compared by *t*-test. Count data were expressed as ratios, and the *χ*^2^ test was used for comparison. *p* < 0.05 was considered statistically significant.

**Table 2:** MMT muscle strength grading criteria.

Grade	Criteria
Grade 0	No signs of muscle contraction
Grade 1	Slight signs of muscle contraction
Grade 2	The range of motion is 1/4 of the normal side
Grade 3	The range of motion is 1/2 of the normal side
Grade 4	Muscle contraction is basically close to normal but slightly asymmetrical to the normal side
Grade 5	Normal muscle contraction and symmetry with normal side

**Table 3:** Cosmetic results evaluation form.

Cosmetic results	Grade	Score	Criteria
Double eyelid incision	Very satisfied	3	Symmetrical, no elimination
	Satisfied	2	Partial disappearance of double eyelid to form asymmetry, but acceptable
	Poor	1	Complete disappearance of double eyelid or unacceptable asymmetry
Eyelid shape	Very satisfied	3	Natural symmetry, the radian is satisfied without peaks
	Satisfied	2	Moderately spiky or flat eyelid, satisfactory to both parent and doctor
	Poor	1	Eyelid appearance needs to be reworked
Double eyelid height symmetry	Very satisfied	3	Bilateral eyelid height difference ≤1 mm
	Satisfied	2	1 mm <Bilateral eyelid height difference ≤2 mm
	Poor	1	Bilateral eyelid height difference >2 mm

## Results

### Comparison of Eyelid Status Between Two Groups in Each Month After Surgery

**Figure 1:**
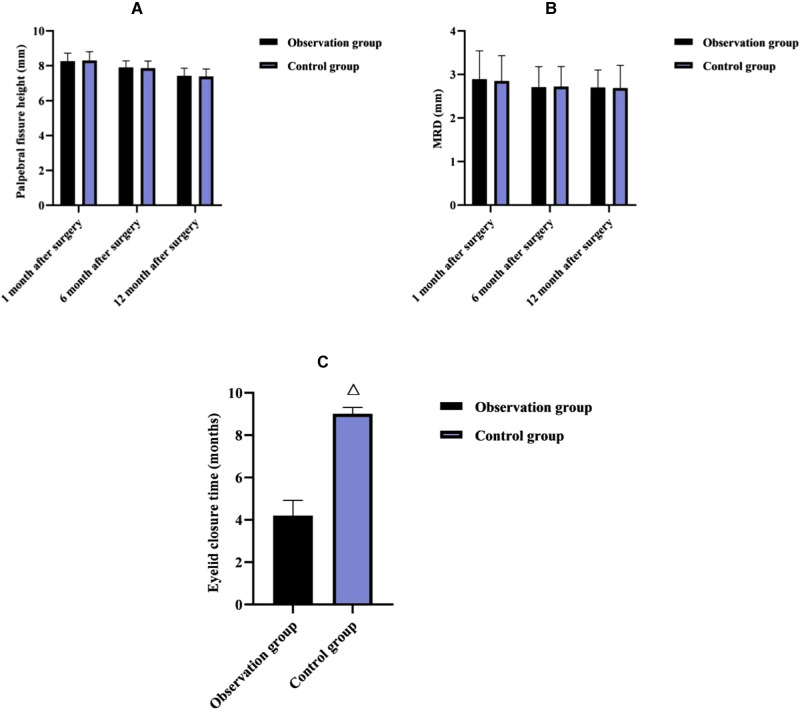
Comparison of eyelid status between two groups in each month after surgery $\left(\bar{x}\pm s\right)$. Note: (**A**) Palpebral fissure height (mm). (**B**) MRD (mm). (**C**) Eyelid closure time (months). Note: Compared with the observation group, ^△^*p* < 0.05.

As shown in **Figure 1**, at 1, 6 and 12 months after surgery, there was no significant difference in terms of palpebral fissure height and MRD between both groups (*p* > 0.05); After surgery, the eyelid closure time was shorter in the observation group than in the control group (*p* < 0.05).

### Comparison of Ocular Surface Status Between Two Groups in Each Month After Surgery

**Figure 2:**
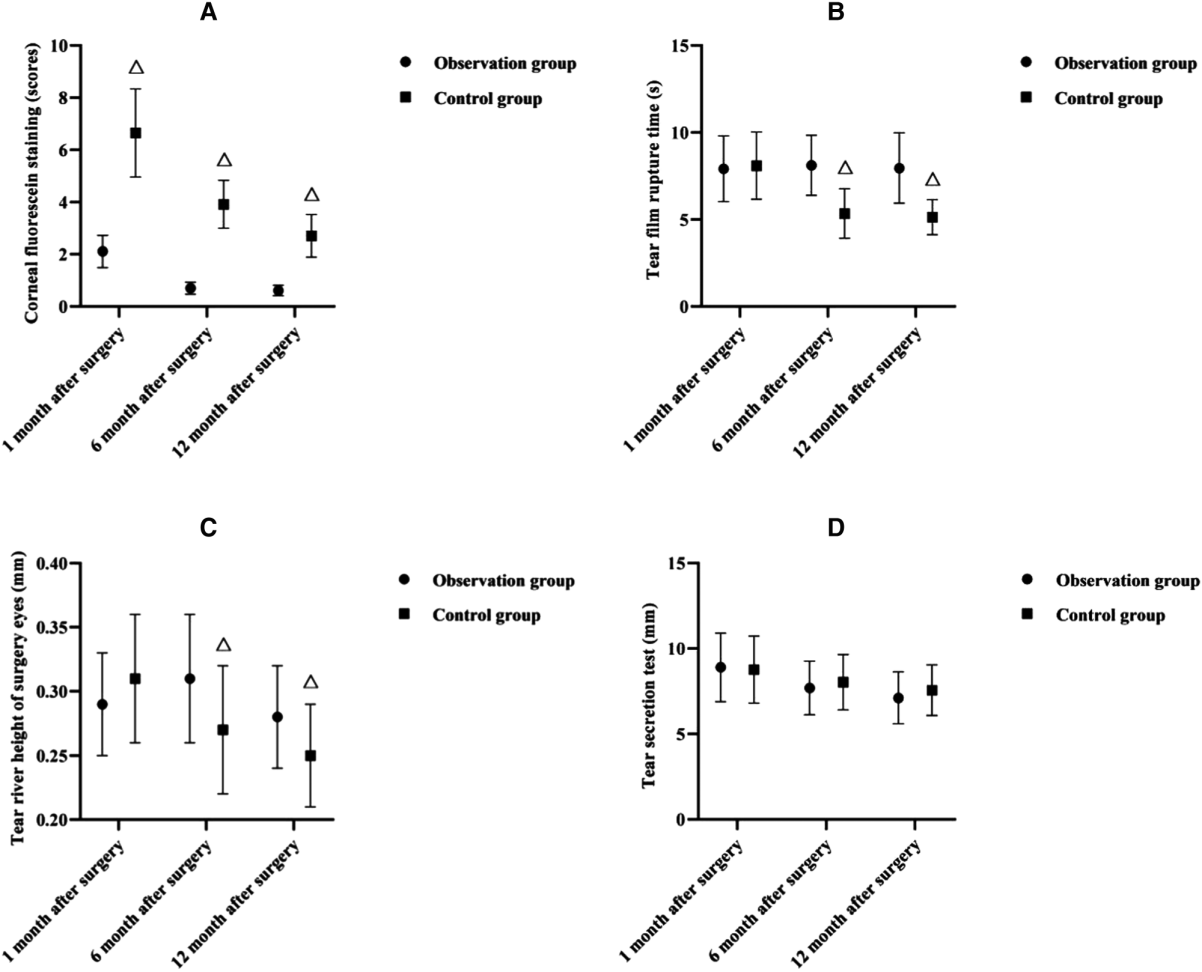
Comparison of ocular surface status between two groups in each month after surgery $\left(\bar{x}\pm s\right)$. Note: (**A**) Corneal fluorescein staining (scores). (**B**) Tear film rupture time (s). (**C**) Tear river height of surgery eyes (mm). (**D**) Tear secretion test (mm). Note: Compared with the observation group at the same time, ^△^*p* < 0.05.

As shown in **Figure 2**, at 1, 6 and 12 months after surgery, the CFS scores were lower in the observation group than in the control group (*p* < 0.05); at 6 and 12 months after surgery, the TBUT was longer and the surgical eye LRH was higher in the observation group than in the control group (*p* < 0.05); at 1, 6, and 12 months after surgery, there was no significant difference in STI between both groups (*p* < 0.05).

### Comparison of Frontal Muscle Strength of Affected Side Between Two Groups in Each Month After Surgery

**Figure 3:**
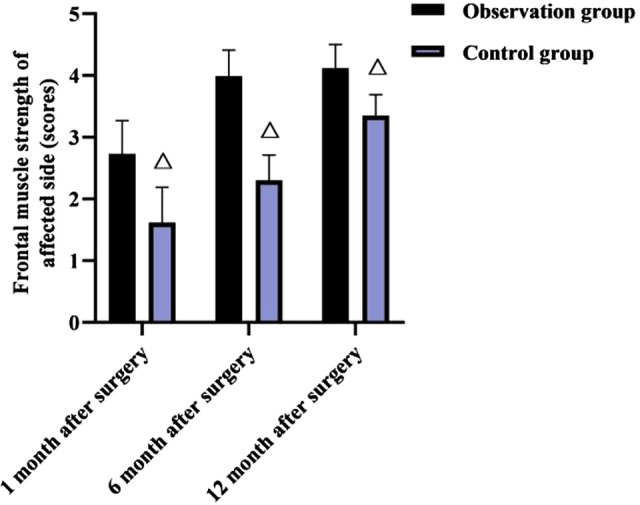
Comparison of frontal muscle strength of affected side between two groups in each month after surgery ($\bar{x}\pm s$, score). Note: Compared with the observation group at the same time, ^△^*p* < 0.05.

As shown in **Figure 3**, at 1, 6 and 12 months after surgery, the frontal muscle strength of affected side was higher in the observation group than in the control group (*p* < 0.05).

### Comparison of Cosmetic Effect Between Two Groups in Each Month After Surgery

**Figure 4:**
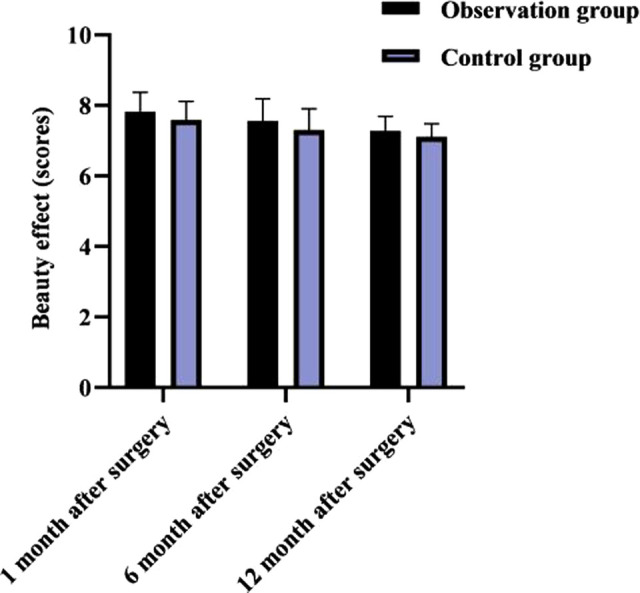
Comparison of cosmetic effect between two groups in each month after surgery ($\bar{x}\pm s$, score).

As shown in **Figure 4**, at 1, 6, and 12 months after surgery, there was no significant difference in cosmetic results between both groups (*p* > 0.05).

### Comparison of Postoperative Complication Rates Between Two Groups

**Figure 5:**
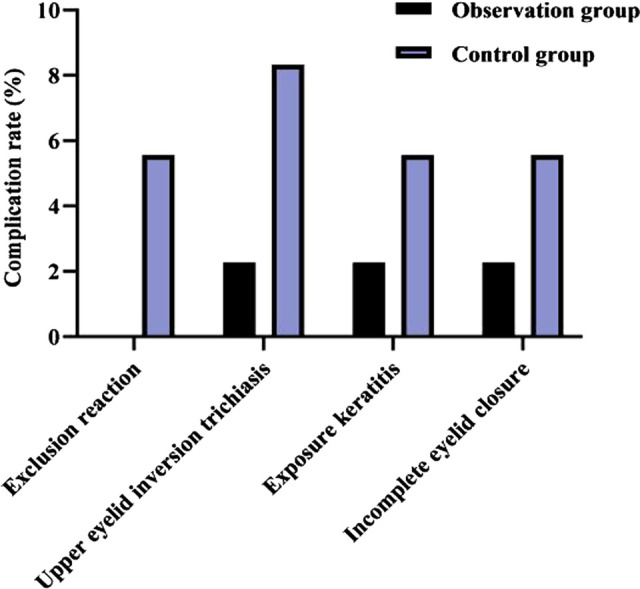
Comparison of postoperative complication rates between two groups (n, %).

As shown in **Figure 5**, after surgery, in the observation group, one case (2.27%) of upper eyelid inversion trichiasis, one case (2.27%) of exposure keratitis, and one case (2.27%) of incomplete eyelid closure occurred; in the control group, two cases (5.56%) of exclusion reaction, three cases (8.33%) of upper eyelid inversion trichiasis, two cases (5.56%) of exposure keratitis, and two cases (5.56%) of incomplete eyelid closure occurred. The overall complication rate in the observation group (6.82%) was lower than that in the control group (25.00%) (*p* < 0.05).

## Discussion

Congenital ptosis, especially moderate and severe ptosis, affects children’s visual function and aesthetics, and also interferes with their normal quality of life (11, 12). Hypoplasia of the levator palpebral is the main pathogenesis in congenital cases, and may also be associated with dysgenesis or insufficiency of the central and peripheral nerves which innervate the levator palpebral (13). Early surgical correction to restore the visual function of children and improve the quality of life of children is the main treatment strategy at present, and the surgical method used has become a hot topic of clinical research. According to consensus (10), for moderate ptosis, because the pupil can be partially or completely exposed, form deprivation amblyopia is less likely to occur and can be surgically corrected with local anesthesia when the child is older, but considering psychosocial factors, surgery is recommended at preschool age, i.e., 3 to 5 years; for severe ptosis in one eye, where the pupil is completely obscured and the child looks with the head up, surgical correction is recommended around 1 year of age to prevent form-deprivation amblyopia and spinal developmental problems. In this study, 80 clinical cases of moderate to severe unilateral congenital ptosis between the ages of 1 and 6 years were selected for comparative analysis; all children had ptosis with poor muscle strength of the levator palpebral and were therefore treated with suspension surgery using the strength of the frontalis muscle.

The present results showed that at 1, 6, and 12 months postoperatively, there was no significant difference in the comparison of palpebral fissure height and MRD between the two groups, but postoperatively, the eyelid closure time was shorter in the observation group than in the control group. It is suggested that e-PTFE frontalis suspension is more effective than frontalis flap suspension in improving eyelid closure and accelerating upper lid function recovery in children with moderate to severe congenital ptosis. It is considered that the e-PTFE material is inherently elastic and allows for moderate contraction and extension of the eyelid, thus reducing the time required for eyelid closure (14). CFS is a direct measure of the state of the ocular surface epithelium (15); TBUT is a measure of tear film stability and is related to tear secretion, tear composition, and ocular surface status (16); LRH is an important indicator to assess the function of tear secretion and should not be less than 0.3 mm normally (17); STI is commonly used for the assessment of tear secretion function and is a test that is closely related to the tear film (18). In this result, at 1, 6 and 12 months after surgery, the CFS scores were lower in the observation group than in the control group; at 6 and 12 months after surgery, the TBUT was longer and the surgical eye LRH was higher in the observation group than in the control group; at 1, 6, and 12 months after surgery, there was no significant difference in STI between both groups. The above indicates that e-PTFE frontalis suspension contributes to a reduction in the degree of postoperative local swelling, and interferes less with the ocular surface status of children with congenital ptosis, but there is no difference in the effect of the two surgical methods on the secretion of tears in children. Considering the reason, the eyelid is easy to swell after frontalis flap suspension in children with congenital ptosis, which leads to difficulty in closing the eyelid, disturbance of blink movement, and dryness and discomfort due to lack of protection of the cornea for a long time; in contrast, after e-PTFE frontalis suspension, the fibroblasts of the child can gradually infiltrate into and around the microporous structure of the e-PTFE patch within 14–28 days after implantation, and their secreted collagen fibrous capsules cover the gaps in the patch, which can avoid swelling of the eyelid to improve the ocular surface status (19).

The frontalis muscle, an important muscle tissue for raising the upper lid, works with the levator muscle to lift the upper lid, and plays an important role in cases of poor levator muscle strength. The frontal muscle flap belongs to the autologous tissue, which is not only rich in blood supply, but also has innervation and strong muscle strength, and because it is interwoven with the surrounding tissues, it is less prone to flap relaxation, which facilitates the ideal effect of frontal muscle flap repair, but can cause some damage to the patient’s frontal muscle strength after surgery (20, 21). The present results showed that the frontal muscle strength of the affected side was higher in the observation group than in the control group at 1, 6 and 12 months after surgery.

The reason for this is considered to be that e-PTFE is relatively lighter in mass, which has less impact on the vascular circulation of the frontalis muscle during the operation, and it is not necessary to remove the suspension material again after the operation, so the damage to the frontalis muscle strength is smaller and the postoperative recovery is better. This result also showed that there was no significant difference between the two groups in comparing the cosmetic results at 1, 6 and 12 months after surgery. This may be due to the fact that the clinical use of the frontalis muscle flap for suspension has good elasticity, high muscle strength, and the operator can adjust the lid curvature according to the actual situation of the child, As a result, the double eyelid curvature after surgery is well formed, the appearance is natural, and the effect is lasting (22–24); Instead, the e-PTFE frontalis suspension surgery uses individualized double eyelid surgery, the surgeon tried to bury the e-PTFE material under the muscle as much as possible during the operation, and the broken end of the material is directly sewn with sutures firmly to increase the flatness, in addition, e-PTFE has excellent biocompatibility and extensibility, stable chemical properties, slow degradation rate, and long retention time, which is conducive to maximizing the postoperative aesthetic effect and long-term maintenance of children.

Traditional frontalis suspension is mostly sutured with mousse thread, which may cause self-rejection as a foreign body, and cause swelling of the eyelid, exposure keratitis, and incomplete closure of the eyelid, which affects the postoperative effect (25). In the present results, the incidences of exclusion reaction, upper eyelid inversion trichiasis, exposure keratitis and incomplete eyelid closure in the control group were higher than those in the observation group, which may be related to the above reasons. It is also suggested that e-PTFE, as a chemical material with high affinity and biocompatibility, has significant advantages in reducing the complications of frontalis suspension and improving the prognosis of children.

## Conclusion

The surgical and cosmetic results of e-PTFE frontalis suspension and frontalis flap suspension applied to congenital ptosis are comparable, but the former has the advantage of faster postoperative recovery, better ocular surface status, less frontali muscle strength damage and fewer complications.

## Data Availability

The original contributions presented in the study are included in the article/Supplementary Material, further inquiries can be directed to the corresponding author/s.
